# A 76‐year‐old male with multiple enhancing brain lesions

**DOI:** 10.1111/bpa.13063

**Published:** 2022-03-10

**Authors:** Kathryn L. Eschbacher, Derek R. Johnson, Sylvia L. Orozco‐Do, Tabinda Jawaid, Audrey N. Schuetz, Aivi T. Nguyen

**Affiliations:** ^1^ 6915 Department of Laboratory Medicine and Pathology Mayo Clinic Rochester Minnesota USA; ^2^ Department of Radiology Mayo Clinic Rochester Minnesota USA; ^3^ 6915 Department of Neurology Mayo Clinic Rochester Minnesota USA; ^4^ Department of Internal Medicine University of Kansas School of Medicine‐Wichita Wichita Kansas USA

**Keywords:** amoebic encephalitis, clinical microbiology, infectious disease, surgical neuropathology

BOX 1 **Slide scan**

Access the whole slide scan at http://image.upmc.edu:8080/NeuroPathology/BPA/BPA‐21‐10‐253.svs/view.apml?

## 1 CLINICAL PRESENTATION

**FIGURE 1:**
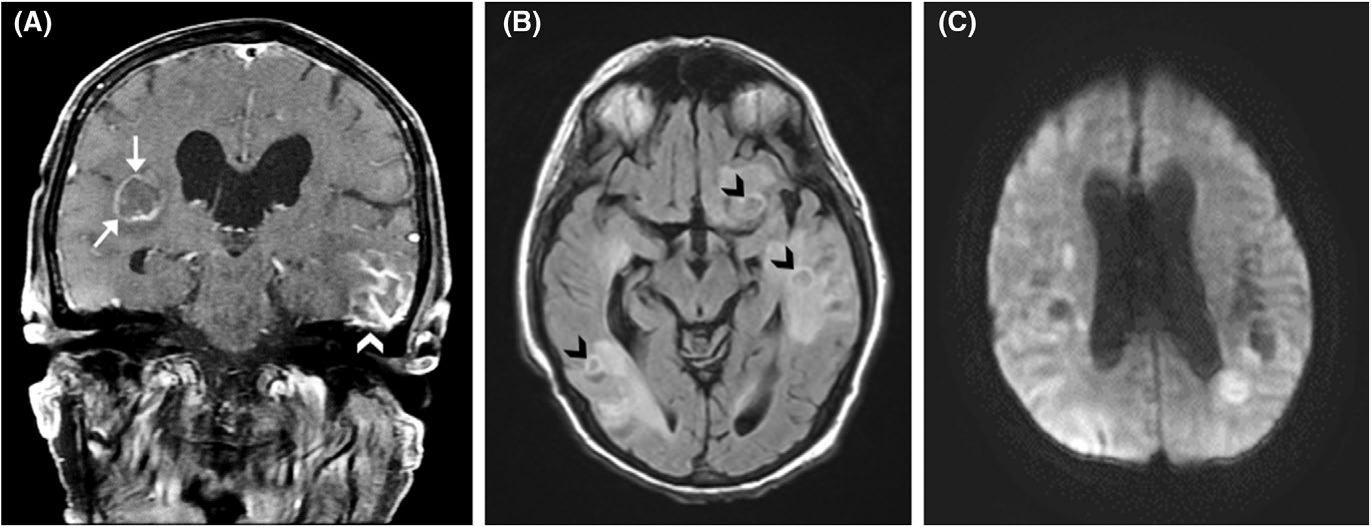
(A) Coronal post‐gadolinium T1‐weighted image demonstrates multifocal enhancement, including both ring‐enhancing lesions (white arrows) and more peripheral cortical involvement (white arrowhead). (B) Axial T2‐weighted FLAIR image displays vasogenic edema surrounding several centrally hypointense lesions (black arrowheads). (C) Axial diffusion weighted imaging (DWI) reveals that the lesions show varying degrees of diffusion restriction, in both solid and peripheral patterns

The patient is a 78‐year‐old male with a history of multiple cutaneous and soft tissue squamous cell carcinomas of the face and ear status‐post resection and a right parotid gland tumor status‐post parotidectomy (diagnosis unknown). He works on a ranch with horses and cows on a property with fresh‐water ponds. He originally presented with flank pain and weakness. Computed tomography of the chest demonstrated soft tissue disease and lymphadenopathy involving the left axilla and anterior mediastinum, bilateral adrenal masses, and a large pleural‐based mass. To evaluate for malignancy, biopsies of a right adrenal nodule and right pleural‐based mass were performed and demonstrated granulomatous inflammation. He was stabilized clinically and discharged. Five months later, he was admitted because of a multiple month history of subacute mental status changes and inability to ambulate. Magnetic resonance imaging of the head demonstrated numerous enhancing intracranial lesions involving both cerebral hemispheres with prominent adjacent edema, the largest measuring 3.6 cm in the right occipital lobe (Figure 1A–C). He was treated with whole‐brain radiation for presumed metastases. *Histoplasma* PCR and serology, tuberculosis PCR, and evaluation for lymphoma were negative.

He continued to decline clinically and underwent an open biopsy of a left frontal brain lesion (Box 1).

**FIGURE 2:**
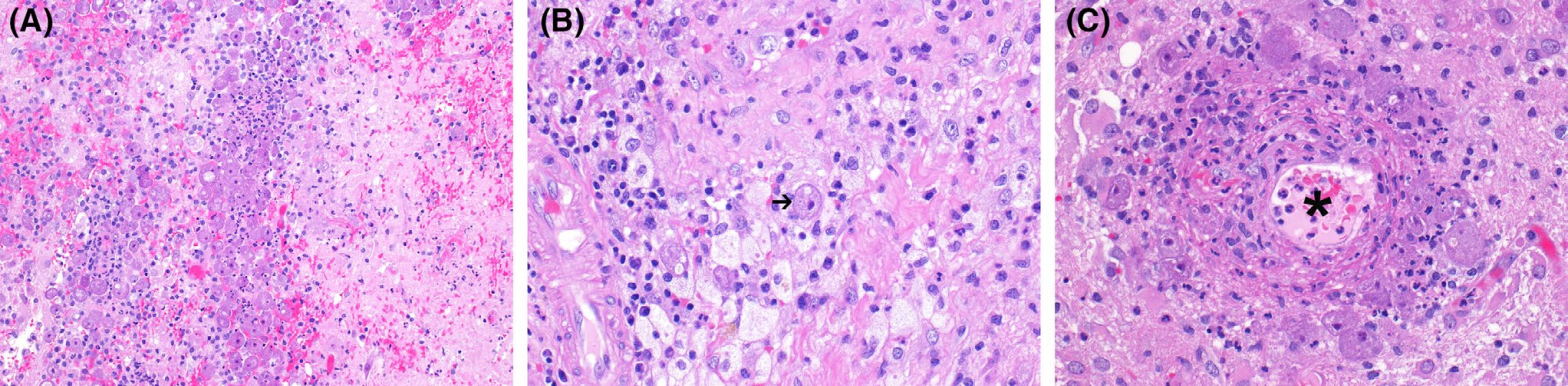
(A) Lower power image of trophozoites in a background of necrotic brain parenchyma and inflammation, H&E 200x. (B) Trophozoite (arrow) with granular cytoplasm and dense karyosome compared to adjacent macrophages with frothy cytoplasm, distinct nuclear contours, and vesicular chromatin, H&E 400x. (C) Trophozoites clustering around a necrotic vessel (asterisk) H&E 400x

Hematoxylin and eosin‐stained sections demonstrated necrotizing encephalitis with numerous associated structures characterized by abundant granular cytoplasm, an indistinct nucleus, and prominent nucleolus‐like structures (Figure 2A–C). These structures were present throughout the brain parenchyma and clustered around blood vessels, which were variably necrotic (Figure 2C). No encysted forms were identified. There was a mixture of associated acute and chronic inflammation and regions of collagen deposition, reminiscent of early granulation tissue formation. A diagnostic study was obtained. Shortly following this diagnosis because of continued patient decline, he was transitioned to comfort care measures and died.

## 2 FINAL DIAGNOSIS

Amoebic encephalitis (granulomatous amoebic encephalitis) because of *Balamuthia mandrillaris*.

The diagnosis was confirmed by multiplex real‐time polymerase chain reaction (PCR) for the detection of free‐living amebae, performed on formalin‐fixed paraffin‐embedded tissue.

## 3 DISCUSSION

Various free‐living amebae (FLAs) can cause central nervous system infection in humans, including *B. mandrillaris*, *Naegleria fowleri*, and multiple species of *Acanthamoeba* [1, 2, 3]. *N. fowleri* leads to a rapidly fatal infection, primary amoebic meningoencephalitis (PAM), which typically affects children and young adults who have recently swam in freshwater [1, 2, 3]. Granulomatous amoebic encephalitis (GAE) is caused by *B. mandrillaris* and multiple species of *Acanthamoeba*. Although patients who develop GAE because of *Acanthamoeba* spp. are typically immunocompromised, *B. mandrillaris* can infect immunocompetent individuals, particularly children and older adults [1, 2]. Systemic infection with *B. mandrillaris* can have a subacute to chronic clinical course, developing over 2 weeks to 2 years [1, 3].

As a soil‐borne pathogen, *B. mandrillaris* is found ubiquitously in the environment, and infection rates have no relation to seasonal changes [2]. Possible routes of infection include inhalation of *B. mandrillaris* present in the soil or inoculation through a wound [3]. The exact pathogenic mechanisms of *B. mandrillaris* that allow it to gain access to the central nervous system are unclear, although studies have demonstrated that it possesses enzymes such as metalloprotease, which may lead to degradation of extracellular matrix (ECM) proteins once it has reached the blood‐brain barrier [2]. By electron microscopy, *B. mandrillaris* has been demonstrated to show cup‐like structures which bind to ECM proteins [2]. Additionally, it has been shown that *B. mandrillaris* induces the production of cytokine interleukin‐6 by brain endothelial cells, which may initiate an inflammatory response [3].

Histologically, two stages within the life cycle of *B. mandrillaris* can be identified: the trophozoite and the cyst form. The trophozoites are typically uninucleate and have a nucleus with a centrally placed, dense karyosome, and abundant bubbly cytoplasm [1, 2, 3]. Although not identified in this case, double‐walled cysts are also often seen and can be a histologic feature that distinguishes it from *N. fowleri* [1, 2, 3]. Trophozoites and cysts may be seen within and surrounding blood vessel walls with an associated mixed acute and chronic inflammation, in addition to hemorrhage and necrosis. Given that patients with GAE may be immunocompromised, the presence of granulomatous inflammation is variable [1].

GAE caused by *B. mandrillaris* may be preceded by painless, plaque‐like skin lesions up to 2 years prior to the encephalitic presentation [1, 2, 3]. However, once GAE has developed, the clinical course is often fatal [2]. Common presenting neurologic symptoms include headache, nausea and vomiting, seizures, meningismus, and myalgia [2, 3]. Imaging findings are variable in patients with GAE, ranging from an isolated to multiple low‐density, “space‐occupying” lesions, which may mimic other disease processes such as abscess, metastatic tumor, or hemorrhage [3].

*B. mandrillaris* is not readily morphologically identifiable from cerebrospinal fluid (CSF) specimens and will not grow on bacteria‐coated agar plates; however, it can be grown on mammalian cell cultures in research settings [1, 2, 3]. CSF studies demonstrate normal to low glucose, increased protein levels, and lymphocytic pleocytosis [2, 3]. Therefore, brain or skin biopsy can be critical to the diagnosis [1]. Moreover, it can be challenging to differentiate between *B. mandrillaris* and *Acanthamoeba* spp. by histology alone. However, they can be differentiated by multiple techniques including immunohistochemistry, indirect immunofluorescence, or multiplex PCR, the latter of which was performed in this case [1, 2, 3].

Although GAE often results in death, rare cases have been reported in which patients have recovered following treatment with a combination of antimicrobials including pentamidine isethionate, sulfadiazine, clarithromycin, fluconazole, and flucytosine [3].

Our case is unusual in that the patient was, to the best of our knowledge, immunocompetent. Given the multifocality of lesions seen on imaging, the clinical suspicion was for a metastatic tumor. Despite the unusual history, the clinical presentation and time course were classic. The time course was likely subacute, as both pleural and adrenal biopsies demonstrated granulomatous inflammation, without definite trophozoites. Upon histologic examination of the brain biopsies and identification of amoeba forms, tissue was sent for multiplex PCR, where the organism identification was confirmed. Ultimately, this case is a classic presentation of an uncommonly encountered form of granulomatous amoebic encephalitis.

## AUTHOR CONTRIBUTIONS

KLE and ATN provided interpretation of pathologic results and study design. KLE and TJ drafted manuscript. DRJ provided neuroradiologic evaluation. SLO provided clinical history. ANS provided clinical microbiology evaluation. KLE and ATN revised and finalized manuscript. All authors edited and approved of the final manuscript.

## ETHICS APPROVAL

The authors have no conflicts of interest to disclose. Internal funding was provided for this project.

## Data Availability

Data sharing is not applicable to this article as no datasets were generated or analyzed during the current study.
